# Physician Pipeline and Pathway Programs: An Evidence-based Guide to Best Practices for Diversity, Equity, and Inclusion from the Council of Residency Directors in Emergency Medicine

**DOI:** 10.5811/westjem.2022.2.54875

**Published:** 2022-07-01

**Authors:** Melissa Parsons, Martina T. Caldwell, Al’ai Alvarez, Dayle Davenport, Moises Gallegos, Adaira Landry, Michael Gottlieb, Sreeja Natesan

**Affiliations:** * University of Florida College of Medicine – Jacksonville, Department of Emergency Medicine, Jacksonville, Florida; † Henry Ford Health System, Department of Emergency Medicine, Detroit, Michigan; ‡ Stanford University College of Medicine, Department of Emergency Medicine, Palo Alto, California; § Rush University Medical Center, Department of Emergency Medicine, Chicago, Illinois; ¶ Harvard Medical School, Department of Emergency Medicine, Boston, Massachusetts; || Duke University Medical School, Department of Emergency Medicine, Durham, North Carolina

## Abstract

Improving the diversity and representation in the medical workforce requires intentional and deliberate efforts to improve the pipeline and pathway for underrepresented in medicine (UIM) applicants. Diversity enhances educational experiences and improves patient care and outcomes. Through a critical review of the literature, in this article we offer evidence-based guidelines for physician pipeline and pathway programs (PP). Recommendations are provided regarding considerations on the types of programs and surrounding implementation to ensure a sound infrastructure and framework. We believe this guide will be valuable for all leaders and faculty members seeking to grow the UIM applicant pool in our efforts to advance diversity, equity, and inclusion within medicine.

## BACKGROUND

Diversity in medicine is an academic imperative. Incorporating diversity offers many benefits to the community, within and outside the walls of the emergency department (ED). 1–3 Diversity creates richer educational experiences, amplifies cultural competencies, and strengthens professionalism. 1,4,5 A diverse physician group also improves patient care and outcomes, 2,6 as physicians underrepresented in medicine (UIM)*1 enhance cultural sensitivity and are more likely to serve low-income, minority, and disadvantaged populations. 2,4,7

Despite the increased recognition and efforts, only a small number of medical specialties have demonstrated statistically significant increases in representation, suggesting that current efforts are insufficient. 8 When the 20 largest specialties in medicine were analyzed between 2007 to 2018, none represented Black or LatinX populations in proportions comparable to the overall United States (US) population. 8 One study found that Black and LatinX representation was still sparse at the medical school faculty level (7.5%), despite 14.1% representation among medical students and 30% at the US population level. 8 Within emergency medicine (EM), women account for only 25% of physicians, while Black, LatinX, and Native American physicians comprise less than 10% of all active emergency physicians. 9 Furthermore, a recent study projects that EM will take about 54 years to reach the level of LatinX faculty representation commensurate with that of the US population. 8

Pipeline and pathway programs (PP)^1^*2 increase opportunities for UIM candidates through outreach, mentorship, and other critical structural support needed to advance diversity, equity, and inclusion (DEI) in medicine. In this paper, we delineate pipeline, pathway, and outreach, mentorship, and other critical structural support needed to advance diversity, equity, and inclusion (DEI) in medicine. In this paper, we delineate pipeline, pathway, and outreach programs, discuss components of PPs, and steps toward successful implementation of DEI initiatives.

## CRITICAL APPRAISAL OF THE LITERATURE

The Council of Residency Directors in Emergency Medicine (CORD) Best Practices Subcommittee publishes a series of articles entitled CORD Best Practice reviews; this manuscript is ninth in the series. 10–17 With the assistance of a medical librarian, we conducted a literature review from inception until January 2021 through MEDLINE via PubMed using the terms and Medical Subject Headings focused on DEI (Appendix). Additionally, a bibliography review was conducted for additional articles. Two reviewers independently screened the 2080 articles, finding 58 relevant for this review.

We created best practice recommendations based on the literature appraisal. Whenever supporting evidence was unavailable, consensus opinion and the authors’ combined expertise were used. The Oxford Centre for Evidence-Based Medicine criteria (Tables 1 and 2) 18 were implemented to provide the level and grade of evidence for each best practice statement. Prior to submission, the manuscript was reviewed by the CORD Best Practices Subcommittee, followed by a two-week review on the CORD website for feedback from the CORD community.

### Barriers to Entering the Health Professions

Barriers exist that hinder students from entering the health professions. Recognition of the barriers (Table 3) for UIM, female, and economically disadvantaged students provides perspective on the challenges that must be overcome to pursue a career in medicine. 19–25 This further highlights the importance of pathway programs to ensure a diverse, equitable and inclusive medical workforce.

Simply recognizing these barriers is not enough. Deliberate actions to mitigate or remove these barriers is imperative, including creation of novel educational and training frameworks that focus on improving trainee performance. 5,26–27 Pathway programs create a supportive network and inclusive culture 26 to reduce the isolation experienced by UIM. The greatest barrier to successful entry into health professions for UIM students is the undergraduate-graduate interface, due to the high attrition rate, with only 38.1% of all UIM doctoral candidates completing their degrees, as opposed to 51.3% of all non-UIM doctorates. 26

### Overview of Pipelines, Pathways, and Outreach Programs

A scoping literature review found the most frequent approaches to increase minority representation in the medical workforce were PPs (43%), changes in affirmative action laws (23%), and changes in admission policies (21%). 5 Pipeline/pathway terminologies are often used interchangeably to describe programs created to increase minority matriculation into medical schools and healthcare workforce diversity. Starting as early as elementary school, these programs offer mentorship to UIM students, 28 offering opportunities to identify and support future diverse medical students. 5,28,29 Outreach programs are typically discrete events, consisting of a single or a select number of time points (Table 4). 3,28,29 They may include second-look visits or weekends for UIM applying to medical schools, often run by the school’s diversity committees or Student National Medical Association groups. 3

Pathway programs effectively enhance academic performance and increase the likelihood that UIM and other disadvantaged undergraduate students enroll in a health professions school. However, the long-term success when targeting high school students remains <20%, suggesting that work still needs to be done to bridge this gap. 28

## TYPES OF PATHWAY PROGRAMS

Pathway programs are a key strategy for increasing the enrollment of UIM students into medical schools. 28 These programs, described below, are created to target different age groups of learners, to provide a variety of educational or developmental benefits,^28^ and to encompass different goals.

### Elementary/High School-to-College Program Pathways

Elementary and high school programs help pre-college UIM students succeed through their primary and secondary education, to continue progressing down the pathway toward medical school. Early introduction and exposure to healthcare has been shown to effectively influence career decisions. 32 Studies have found that among all ethnic groups, the major hurdles to attending medical school are obtaining a high school diploma and bachelor’s degree. 33 Once this hurdle is overcome, the proportion of UIM college graduates to apply to medical school are similar to proportions of White college graduates. 33

Goals of these programs include preparing students for college life, 27,34 exploring different healthcare careers,^27^,^35^–^37^ increasing research exposure, 38–39 and improving basic science knowledge. 2 Programs vary from summer experiences 2,37,40 to longitudinal experiences during the school year 19 and offer educational components, such as core sciences (eg, biology, chemistry) 2 or healthcare-related topics (eg, disparities, physiology, patient interview sessions). 19,27,32,41,42 Interactive sessions such as simulation, physical exam practice, workshops, and seminars are also included. 19,31,32,36,41,43

College preparation is a significant component of many elementary-to-high-school PPs, and include exposure and guidance to the university admissions process, 2,19,41 financial aid guidance, 2,19,41 and college fairs. 43 Because standardized test scores continue to be a barrier for UIM, Scholastic Assessment Test preparation is also a frequent element of these programs. 2,39,44

Mentorship is a crucial component of elementary-to-high-school PPs and may be provided by medical students, graduate students, or working professionals. 2,29,37,41,45 Shadowing opportunities are important, 2,35 including patient care in free clinics. 42 Multiple studies suggest that educational content and mentorship can be provided by medical students. 32,46 Another strategy was the creation of Health Professions Affinity Clubs, in which volunteers visited high schools to introduce students to health professions via projects, mentoring, and shadowing opportunities. 29

### College-to-Medical-School Pathway

Undergraduate level pathways serve to expose UIM students to the health professions. The structure of these programs vary in length, 30,31 frequency of meetings, 47 time of year, 39 and depth of contact. 47 Another common PP is summer internship programs offering opportunities in education, 27 research, 39,47 clinical care, 48 or a combination of these (Table 5). 48–51

Longitudinal pathways exist to offer “living and learning communities,” consisting of a designated dorm floor for pre-health students to enhance networking and surround undergraduate students with a supportive cohort. 47 Undergraduate pre-health organizations also provide career counseling, test support, networking opportunities with health professions students and faculty, and shadowing opportunities. 47

### Bachelor of Science-Medical Doctor (BS-MD) Pathway

The combined BS-MD program can be a high school or undergraduate school to medical school pathway. For example, the Premedical Honors College, an eight-year college-to-medical-school program targeting South Texas medically underserved counties, 52 of which the majority of the population (81%) identifies as LatinX. It provides conditional acceptance to medical school based on the completion of the bachelor of science and includes rigorous undergraduate curriculum, enrichment experiences, clinical experiences, tutoring, and a summer research program. 52 It has successfully produced 134 medical school matriculations, 110 (82%) of which are UIM and 106 (79%) are LatinX. 53

Other BS-MD programs offer undergraduates conditional acceptance to medical school. The Mount Sinai School of Medicine created a Humanities and Medicine program, an early admissions program that allows sophomore-year undergraduate students to apply and pursue their interests in humanities and social sciences prior to matriculating to medical school. 39 These programs often do not require Medical College Admission Test (MCAT) scores and thus remove one barrier to gain acceptance to medical school. 54

### Community Colleges (CC) to Medical School

Another undergraduate-to-medical-school route is the community college (CC) pathway. Defined as two-year post-secondary education institutions, CCs serve as a common pathway to the attainment of higher education for low-income and UIM students. Talamantes studied medical students’ educational path to better understand the use of CCs, 54 and found that of students using a CC pathway, LatinX were the most common racial-ethnic group (34%), followed by Black (28%), White (27%), and Asian (27%) matriculants. 54 This data suggests that PPs targeting CCs may be a promising approach to increasing the diversity of medical students. An improved process to transfer credits from CCs to four-year institutions is recommended.^55^

### Post-Baccalaureate Pathway Programs

Post-baccalaureate pathway programs (PBPP) are an important strategy for increasing diversity among medical school matriculants. The UIM and disadvantaged students tend to be at greater risk for academic difficulties and lower MCAT scores, which are known barriers to medical school admission. 35,56 The PBPPs frequently involve a one- or two-year curriculum 38,56,57 that emphasizes basic science skills required for the MCAT and medical school. Other topics include academic enrichment skills, personal well-being, and professionalism. 31,35,38,57–59 Some PBPPs may offer research options, 35,38,39 while others focus on clinical opportunities with underserved patients. 51 These PBPPs consist of small cohorts (2–8 students) and are often supported through institutional funding to provide financial support and tuition waivers. 31,38

In 2014, 36% of national PBPPs identified themselves as having a special focus on UIM or economically or educationally disadvantaged students. 58 Many programs offer early or conditional acceptance to degree-confirming MD, PhD, and combined MD/PhD programs, pending successful completion of the program. 2,31,35,39,51,52,58 A few PBPPs confer master’s degrees or certificates upon successful completion. 2,51,56 Academic or civic credit may also be awarded to students for participating. 34,48 Students are selected via a national open application, 38 with preference often given to those who had been unsuccessful in their medical school applications. 31,51

Despite having academic profiles that were not promising for medical school admissions on entering the Medical/Dental Education Preparatory Program (MEDPREP) program, 83.3% of graduates successfully matriculated in medical school. 56 Of those, 53% worked in primary care and 40% worked in medically underserved areas after graduation. 56 Long-term data has shown that PBPP graduates have pursued careers in every specialty and are more likely to provide care in underserved areas or for vulnerable populations. 28

### Historically Black Colleges and Universities

Historically black colleges and universities (HBCU) and historically black medical schools (HBMS) have a significant impact on the diversity of medicine. 61,62 The HBMSs are instrumental in the overall representation of Black chairs, faculty, and students in US medical schools. 62 Xavier University and Howard University are the top two producers of Black graduates of medical schools. 63 Black students who graduate from HBCUs were found to be more likely to go to graduate school and complete their doctoral degrees than Black students from other schools. 61

The HBCUs were found to devote greater effort to premedical training, developing strong relationships with medical schools and offering a range of sponsored enrichment opportunities to their students. 63 Successful interventions include providing all premedical students a core curriculum instead of allowing them to choose their courses, providing tutors for all first- and second-year students, and beginning MCAT practice during their first year of college. 61 The HBCUs are also successful in building strong pathway partnerships with medical schools, educating on health disparities, and teaching cultural competency skills. 61 Best practice recommendations are summarized in Box 1.

Box 1: **Best Practice Recommendations**

**Table:** 

**Interventions should be focused on helping to overcome the major hurdles to medical school entrance for UIM (e.g., high school diploma and a bachelor’s degree). (Level 5, Grade D)** **Develop and support PPs to create opportunities for the introduction and exposure to healthcare at an early time point in order to influence career decisions in UIM. (Level 4, Grade C)** **Allow medical students to deliver educational content and mentorship for PPs and outreach programs.(Level 5, Grade D)** **Consider PPs targeting community colleges as an approach to increasing the diversity of medical school applicants, most notably Latinx UIM. (Level 5, Grade D)** **Consider post-baccalaureate premedical programs with a focus on UIM or disadvantaged students as an important strategy to increasing diversity in medical school matriculants. (Level 5, Grade D)** **Collaboration with HBCUs is beneficial, as HBCUs graduate students that are more likely to attend and complete graduate school. (Level 5, Grade D)**

*UIM*, Underrepresented in Medicine; *PP*, Pathway programs; *HBCU*, Historically Black Colleges and Universities

## INFRASTRUCTURE AND FRAMEWORKS FOR IMPLEMENTING PATHWAY PROGRAMS

Factors related to implementing PPs are important determinants of the success of these programs. Below, we explore the following implementation elements: frameworks and theories; funding; participant selection; academic enrichment and instructional design; and mentoring, advising, and networking.

### Frameworks and Theories

**Figure 1:**
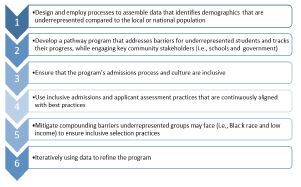
Young and colleagues’ framework for diversity-related pathway programs.

Several articles outlined PPs’ frameworks and theoretical underpinnings. Young built on the knowledge translation framework to generate a six-part framework for developing PPs (Figure 1). 64 When developing a portfolio of comprehensive PPs across the educational continuum, Grumbach suggested that institutions adopt a “distal-to-proximal” strategy to prioritize later-stage participant support (eg, post-baccalaureate programs) and then work backwards to include earlier stage programs. 65

Johnson and Bozeman constructed the asset bundles model from other models and theories that focus on human capital (eg, knowledge and technical abilities), social capital (eg, ability to tap into resources embedded in relationships), and the ways in which institutions perpetuate marginalization. 25 Asset bundles are “the specific sets of abilities and resources that individuals develop that help them succeed in educational and professional tasks.” 25 The authors assert that these five asset bundles are critical to retaining UIMs on successful educational pathways (Table 6). 25 Many PPs incorporate individual assets, but few programs work to enhance all the assets. 25,49

### Funding

Sustainable funding is critical for the success and survival of PPs. Programs are usually funded from multiple sources, including federal, foundation, and institutional investments. 27,29,31,34,37,38,41,44,48,50,57 Less commonly, programs received funding from non-profit professional organizations, private entities, and state legislative appropriations, 36,38,41,44,47,57 or program alumni efforts. 57 Federal funding for PPs has been dramatically reduced over the years. 65 In drafting this manuscript, we searched for several of the federal funding sources for cited programs, which currently are not taking new applications. As external funding for PPs shrink, greater onus is on universities and health systems to fund these initiatives.

The program budgets ranged widely from $2,600 (2007 dollars) for a student-run, specialty-specific initiative, to $25,000 (2018 dollars) for a two-day workshop, to several million dollars for a comprehensive collection of PPs. 30,34,47,49,65 The University of Illinois at Chicago’s Urban Health Program is funded by the state and seven university colleges to amass an approximately $4.3 million budget (2012 dollars) that serves hundreds of preschool through graduate school students annually. 47 The state’s financial contribution was tied to metrics that demonstrated success in supporting UIM students matriculation to the health professions. In 2011, highly effective PBPPs typically cost $20,000.^65^ Those aimed at UIM scholars tended to discount fees for students and rely mostly on institutional sources of funding. 52 Some programs offered students stipends, scholarships, and/or wages to cover the costs of travel, tuition, fees, attendance at conferences and workshops, and other financial needs. 2,19,27,31,38,40,49,52,58

### Participant Selection

Most PPs aim to support and facilitate educational and healthcare career advancement for UIM racial and ethnic groups. 1 9,27,30,31,35,38,41,47–50,52,56–58,60,66,67 Few programs share their participant selection criteria in detail and rarely state race/ethnicity criteria. 30 Commonly, programs used proxy criteria for race/ethnicity including being from educationally/economically disadvantaged backgrounds. 27,30,31,35,38,47,48,66 To attract UIM students, some PPs recruited from majority UIM schools and communities, focused on racial/ethnic health inequities, or selected students whose attributes and interests reflected the institution’s mission. 27,31,34–36,38,39,41,44,48–50,52,58

Few programs stated they used holistic review to select participants. 31,41,47,58 Traditional measures of academic success (ie, grades and test scores) were only occasionally included as selection criteria. 2,19,36,38,40,49,50,52,56,67 One program only used academic measures to exclude candidates with extremely low scores, 48 while another program intentionally sought candidates whose academic performance may not have matched their potential. 38 Nevertheless, satisfactory test scores and grades were used as measures of successful completion of the program. 31,35,52,58

While most programs included interest in medical or science careers in their selection, 2,19,27,30,35,44–46,50 one program specifically excluded students who had previously shadowed physicians or participated in medical-related community service in order to capture students who needed an initial exposure to medicine. 42 Most programs only required written application materials, but the MEDPREP program also required on-site reading comprehension testing and two faculty interviews. 56 The MERIT program invited students to a three-week “tryout” medical leadership course, evaluating students based on peer interactions, homework, and punctuality as indicators of their passion and potential, as opposed to traditional academic measures. 41 Note that this program made a significant and longitudinal investment in their participants for seven years, prompting their intensive screening process. A detailed target population resource based on target population, selection criteria, and application components can be seen in the Appendix.

### Academic Enrichment and Instructional Design

The central feature of PPs are educational support and skills development. Many provided math and science enrichment and test prep through locally developed programs or professionally delivered courses 2,27,30,31,38,39,41,44,47,52,56–59,69 with several programs developing individualized focused educational plans. 25,27,30,35,38,57,58 Other academic enrichment activities include one-on-one and group tutoring, study skills, critical thinking, leadership skills, public speaking, and writing. 2,27,31,38,39,47,49,52,56–59,66–68 A few programs taught professionalism skills such as punctuality, email writing, goal-setting, “appearance,” “etiquette,” and “speaking and dressing appropriately.” 40,41,58 Some programs hosted wellness sessions and stress reduction techniques to mitigate burnout. 27,41

Academic enrichment was provided in both didactic and experiential formats, using large- and small-group formats and employing multiple educational approaches (Table 7). Clinical shadowing opportunities with faculty and resident physicians were included in many programs. 2,31,35,36,41,42,47,49–52 These shadowing opportunities often progressed to shadowing with history-taking and ended with independent history-taking and oral presentations. 51

### Mentoring, Advising, and Networking

Mentorship helps to transform students’ thinking, enhance knowledge, develop technical skills, broaden aspirations and confidence in a future scientific career, and improve “professional socialization.” 68 Although the characteristics students valued in a mentor varied based on the students’ demographics, all agreed that engaged mentors were the most effective. The importance of concordant mentors (racial/ethnic, gender, and sexuality) was also underscored in several studies, citing the intangible benefit of having a mentor that “looks like you” in helping students visualize themselves as successful physicians. 26,27,38,69,70 Having a concordant mentor may minimize beliefs that their aspirations are unattainable and mitigate experiences of isolation that are linked to low self-efficacy. 25,26,39,31,70

Mentorship may be structured, small group-led faculty or one-on-one mentoring.^27^,^30^,^31^,^34^–^36^,^38^,^41^,^49^,^56^,^66^ Rarely, mentoring and coaching was longitudinal, including after participants complete the program and during major transitions (eg, from high school to university). 37,38,41,49 Peer and near-peer mentoring and advising was also a component of some programs.^36^,^38^,^45^,^47^–^50^,^53^,^56^,^68^.72

In addition to formal mentoring, PPs also facilitate networking opportunities in small groups for students to interact with physicians, scientists, medical graduate students, and alumni. 30,31,36,38–41,43,47,50,66 Often, formal networking occurs over scheduled lunches. Informal networking occurred during events like career fairs, didactics, research symposium, shadowing, and barbecues. Several programs also offered mental health counseling and other intensive social and emotional support to their participants. 2,27,38,47,56–58 Several programs provided general career advising, covered college and medical school admissions, coached students on interview preparation, and counseled on financial planning and scholarships. 2,19,20,30,31,34,38,41,46,47,49,50,56,66,68,71 Best practice recommendations are summarized in Box 2.

Box 2: **Best Practice Recommendations**

**Table:** 

**PPs should use frameworks and theories that leverage participants’ assets, incorporate diverse and developmentally appropriate learning techniques, maximize relevance local health concerns, and center participants’ identities and lived experiences in an affirming way. (Level 5, Grade D)** **PPs should develop robust, intra- and interinstitutional partnerships to ensure success. (Level 4, Grade C)** **Federal, foundation, institutional, and private funding is critical and should be sought out and advocated for. (Level 4, Grade C)** **When creating a program to support UIM groups, clearly state selection criteria including, but not limited to, UIM race/ethnicity selection criteria, along with other primary selection criteria (eg, factors associated with systemic disadvantage, interest in healthcare, markers of academic success). (Level 4, Grade C)** **Create programs for academic enrichment that utilize a variety of approaches and instructions for both didactic and experiential learning. (Level 4, Grade C)** **Programs should consider identity-concordant mentoring, coaching, and networking as they are powerful mechanisms to encourage and motivate UIM success. (Level 4, Grade C)**

*UIM*, Underrepresented in Medicine; *PP*, Pathway programs

## LIMITATIONS

This paper focused on pipeline, pathway, and outreach programs. Recognizing the vastness of DEI, other topics (eg. faculty recruitment and retention, holistic review, mitigating bias in residency recruitment) will be covered elsewhere. It is possible that we may have missed some relevant articles in our search. To mitigate this, a comprehensive search strategy with the aid of a medical librarian was conducted, supplemented by bibliographic review and additional recommendations from topic experts. Much of the research on DEI is observational, and multicenter RCTs are often lacking. Our findings may represent associations as opposed to causation given the nature of the research available. Finally, much of the literature of pathways focuses on general fields in health professions education; there is limited literature specifically focused on pathways within EM.

## CONCLUSION

Pathway programs are critical to increasing diversity within medical schools. Increasing diversity in medical schools is critical to increasing diversity in EM and other specialties. This paper summarizes components of PPs and steps toward successful implementation through best practice recommendations. We hope this manuscript will inform readers on how best to form and sustain new PPs at their institutions.

## Supplementary Information



## Figures and Tables

**Table 1:** Oxford Centre for Evidence-Based Medicine levels of evidence.^17^

Level of evidence	Definition
1a	Systematic review of homogenous RCTs
1b	Individual RCT
2a	Systematic review of homogenous cohort studies
2b	Individual cohort study or a low-quality RCT*
3a	Systematic review of homogenous case-control studies
3b	Individual case-control study**
4	Case series/Qualitative studies or low-quality cohort or case-control study***
5	Expert/consensus opinion

* defined as <80% follow up;

** includes survey studies and cross-sectional studies;

*** defined as studies without clearly defined study groups.

*RCT*, randomized control trial.

**Table 2:** Oxford Centre for Evidence-Based Medicine grades of recommendation.^17^

Grade of evidence	Definition
A	Consistent level 1 studies
B	Consistent level 2 or 3 studies or extrapolations* from level 1 studies
C	Level 4 studies or extrapolations* from level 2 or 3 studies
D	Level 5 evidence or troublingly inconsistent or inconclusive studies of any level

* ”Extrapolations” refer to data used in a situation that has potentially clinically important differences from the original study situation.

**Table 3:** Barriers to health professions education for students underrepresented in medicine.^13^,^18^–^23^

Socioeconomic barriers	High indebtedness Lack of encouragement to complete high school, enter college, or pursue higher education Lack of financing for college and graduate school Low income/poverty Need to provide financially for family Teen pregnancy/Early parenting
Educational barriers	Concerns regarding duration of training High dropout rates of UIM in high school and college Hidden curriculum propagating bias/racism Less developed networks and lack of mentorship Lack of minority faculty Lack of traditional educational path Low on-time graduation rates Poor performance on standardized achievement tests
Psychosocial barriers	Difficulties in acclimation to majority culture A sense of isolation due to low visibility of others with similar backgrounds Lack of support from family and friends Lack of cultural representation Stereotype threat/risk of person conforming to stereotypes about their social group Undesirable geographic distance of school from student’s home and community Low expectations of academic ability by others

*UIM*, underrepresented in medicine.

**Table 4:** Outreach activities for students underrepresented in medicine.^28^,^29^

Workshop on getting admitted to medical school MCAT guidance Workshop on writing a personal statement Education on financing medical school Mock interviews Shadowing opportunities such as “Day in the Life of a Medical Student.” Sample lectures, labs, and small-group discussions Second look visits or weekends for UIM applying to medical schools

*MCAT*, Medical College Admission Test; *UIM*, underrepresented in medicine.

**Table 5:** Examples of undergraduate pathway programs.

Name	
Summer Medical/Dental Education Program^2^,^24^	National summer enrichment program for college undergraduates from disadvantaged backgrounds that provides intensive preparation for medical or dental school.^2^,^25^ Provides courses in science, math, writing, and career development skills based on an individualized education plan.^25^ Medical school acceptance rate of 64% among undergraduate participants.^2^
Health Frontiers in Tijuana Undergraduate Internship Program^41^	14 consecutive. weekly, one-hour clinical shadowing engagements. Integrates US undergraduate students longitudinally in a US-Mexico binational free clinic alongside their Mexican undergraduate peers. Exposes undergraduate interns to clinicians with different health careers based on student’s area of interest or at the medical student-run free clinic. Incorporates education on medical Spanish, conditions seen frequently in clinics, and barriers to healthcare.^41^
SEALS^42^	Six-week program that promotes socialization, education in science learning, acquisition of financial literacy, leveraging of mentorship and networks, and resilience Sessions use lectures, dissection lab, clinical shadowing, workshops on writing skills, and workshops on professional development.^42^
Health Disparities Clinical Summer Research Fellowship Program^47^	Incorporates healthcare exposure with research. Community organization that involves shadowing healthcare professionals, engaging in enrichment activities, and providing information on health-professions graduate school admissions, as well as preparing for the MCAT.

*US*, United States; *MCAT*, Medical College Admissions Test.

**Table 6:** Asset-bundle model components and descriptors.

Asset bundle	Description
Human Capital: Educational Endowments	Focuses on academic performance, which is primarily determined by students’ high school math and science curricula and teacher quality. Advance courses such as advanced placement, international baccalaureate, and college prep, as well as hands-on laboratory experiences, study groups, tutors, and systemic educational reform to improve math and science curricula can enhance this asset.
Human Capital: Science Socialization	UIM students may need additional encouragement to envision themselves as physicians and scientists, as it is unlikely that they have regular access to role models in these fields in their homes or proximal communities due to systemic underrepresentation. This can be done by 1) emphasizing the relevance of science and technology to addressing problems in their community; 2) exposing students to successful identity-concordant scientists and physicians; and 3) developing individualized plans to benchmark students’ progress toward their career goals.
Social Capital: Network Development and Expansion	Mentoring and extracurricular activities are important avenues to develop and expand social networks. Mentoring that is both identity-concordant and cross-cultural can be successful in expanding students’ networks and facilitating positive career outcomes. Broadening peer networks through multiracial study groups, for instance, may expose UIM students to information and resources they would not otherwise obtain.
Social Capital: Family Expectations	Family expectations, which may be dictated by constraining social norms such as women in caretaking roles, may create tension with educational goals. Conversely, familial expectations that affirm educational goals can be a positive influence. These dynamics are difficult to impact externally; thus, programs may need to reinforce other assets such as science socialization.
Financial Capital: Material Resources	Scholarships and grants are critical resources needed to reduce education attrition among students who do not have significant familial financial resources. Economically disadvantaged students often take part-time employment, limiting their time for academic study and extracurricular enrichment, which further limits their competitiveness for scholarships.

*UIM*, underrepresented in medicine.

**Table 7:** Structured learning approaches for pathway programs.^2^,^11^,^13^–^16^,^25^,^28^,^29^,^31^–^35^,^38^,^41^–^44^,^50^,^56^–^58^,^60^

Lectures and seminars
Readings
Videos
Clinical vignettes
Problem-based learning
Hands-on dialectics
Inquiry-based lab experiments
Simulation training
Facilitated review
Role-playing
Skits
Debates on medical ethics
Games
Props and models
Interviewing standardized patients
Personal narratives
Written reflections
Humor

## Footnotes

1* The Association of American Medical Colleges (AAMC) defined the term underrepresented minority (URM) to reflect the racial groups of Black, Mexican American, mainland Puerto Rican, and Native American (American Indian and natives of Alaska and Hawaii). In 2003, to encompass the racial and ethnic populations within medicine who are underrepresented when compared to their respective numbers in the context of the greater population, this was further clarified to “underrepresented in medicine”(URiM or UIM). Although we recognize the use of URiM or UIM interchangeably, for consistency, we will use the term UIM throughout this paper.

2* The words pipeline and pathway carry with them different perspectives. Pipeline can be considered restrictive, exclusive, and confining and may have potentially harmful attributions, such as the “school-to-prison” pipeline.^10^ Pathways, in contrast, acknowledges a compendium of entry points that support the program. Because of this, some advocate for the move away from the word pipeline to favor the use of pathway,^10^ and thus pathway will be used throughout the remainder of this article.

## CORD Best Practice Committee 2020–2021

- **Michael Gottlieb, MD – Co-Chair**
  *Rush University Medical Center*
- **Sreeja Natesan, MD – Co-Chair**
  *Duke University*
- **John Bailitz, MD**
  *Northwestern University, Feinberg School of Medicine*
- **Brian Barbas, MD**
  *Loyola University Medical Center*
- **Jennie Buchanan, MD**
  *Denver Health Medical Center*
- **Richard Byyny, MD**
  *Denver Health Medical Center*
- **Guy Carmelli, MD**
  *University of Massachusetts Medical School*
- **Molly Estes, MD**
  *Loma Linda University*
- **Katja Goldflam, MD**
  *Yale University*
- **Andrew Grock, MD**
  *University of California - Los Angeles*
- **Jaime Jordan, MD**
  *University of California - Los Angeles*
- **Deena Khamees, MD**
  *University of Michigan*
- **Andrew King, MD**
  *The Ohio State University*
- **Krystin Miller, MD**
  *The Ohio State University*
- **Melissa Parsons, MD**
  *University of Florida-Jacksonville*
- **Alexander Sheng, MD**
  *Boston Medical Center*
- **Brian Wood, MD**
  *St. Joseph’s Medical Center*

## References

[1] Heron SL, Lovell EO, Wang E (2009). Promoting diversity in emergency medicine: summary recommendations from the 2008 Council of Emergency Medicine Residency Directors (CORD) Academic Assembly Diversity Workgroup. Acad Emerg Med Off J Soc.

[2] Smith SG, Nsiah-Kumi PA, Jones PR (2009). Pipeline programs in the health professions, part 1: preserving diversity and reducing health disparities. J Natl Med Assoc.

[3] Rumala BB, Cason FD (2007). Recruitment of underrepresented minority students to medical school: minority medical student organizations, an untapped resource. J Natl Med Assoc.

[4] Pierre JM, Mahr F, Carter A (2017). Underrepresented in medicine recruitment: rationale, challenges, and strategies for increasing diversity in psychiatry residency programs. Acad Psychiatry.

[5] Kelly-Blake K, Garrison NA, Fletcher FE (2018). Rationales for expanding minority physician representation in the workforce: a scoping review. Med Educ.

[6] Sanson-Fisher RW, Williams N, Outram S (2008). Health inequities: the need for action by schools of medicine. Med Teach.

[7] Shipman SA, Wendling A, Jones KC (2019). The decline in rural medical students: a growing gap in geographic diversity threatens the rural physician workforce. Health Aff Proj Hope.

[8] Bennett CL, Yiadom MYAB, Baker O (2021). Examining parity among Black and Hispanic resident physicians. J Gen Intern Med.

[9] Pierce AE, Moreno-Walton L, Boatright D (2020). Advancing diversity and inclusion: an organized approach through a medical specialty academy. AEM Educ Train.

[10] Gottlieb M, King A, Byyny R (2018). Journal club in residency education: an evidence-based guide to best practices from the Council of Emergency Medicine Residency Directors. West J Emerg Med.

[11] Estes M, Gopal P, Siegelman JN (2019). Individualized interactive instruction: a guide to best practices from the Council of Emergency Medicine Residency Directors. West J Emerg Med.

[12] Chathampally Y, Cooper B, Wood DB (2020). Evolving from morbidity and mortality to a case-based error reduction conference: evidence-based best practices from the Council of Emergency Medicine Residency Directors. West J Emerg Med.

[13] Parsons M, Bailitz J, Chung AS (2020). Evidence-based interventions that promote resident wellness from the Council of Emergency Residency Directors. West J Emerg Med.

[14] Natesan S, Bailitz J, King A (2020). Clinical teaching: an evidence-based guide to best practices from the Council of Emergency Medicine Residency Directors. West J Emerg Med.

[15] Wood DB, Jordan J, Cooney R (2020). Conference didactic planning and structure: an evidence-based guide to best practices from the Council of Emergency Medicine Residency Directors. West J Emerg Med.

[16] Davenport D, Alvarez A, Natesan S (2022). Faculty recruitment, retention, and representation in leadership: an evidence-based guide to best practices for diversity, equity, and inclusion from the Council of Residency Directors in Emergency Medicine. West J Emerg Med.

[17] Gallegos M, Landry A, Alvarez A (2022). Holistic review, mitigating bias, and other strategies in residency recruitment for diversity, equity, and inclusion: an evidence-based guide to best practices from the Council of Residency Directors in Emergency Medicine. West J Emerg Med.

[18] Phillips R, Ball C, Sackett D 2009 Oxford Centre for Evidence-Based Medicine: Levels of Evidence March 2009.

[19] Roche R, Manzi J, Ndubuizu T (2020). Self-efficacy as an indicator for success in a premedical curriculum for underrepresented minority high school students. J Med Educ Curric Dev.

[20] Odom KL, Roberts LM, Johnson RL (2007). Exploring obstacles to and opportunities for professional success among ethnic minority medical students. Acad Med.

[21] Kumar V, West DL (2019). Bridging the equity gap. Am J Roentgenol.

[22] Freeman BK, Landry A, Trevino R (2016). Understanding the leaky pipeline: perceived barriers to pursuing a career in medicine or dentistry among underrepresented-in-medicine undergraduate students. Acad Med.

[23] Sánchez JP, Castillo-Page L, Spencer DJ (2011). Commentary: the Building the Next Generation of Academic Physicians Initiative: engaging medical students and residents. Acad Med.

[24] Murray-García JL, García JA (2002). From enrichment to equity: comments on diversifying the K-12 medical school pipeline. J Natl Med Assoc.

[25] Johnson J, Bozeman B (2012). Perspective: adopting an asset bundles model to support and advance minority students’ careers in academic medicine and the scientific pipeline. Acad Med.

[26] Allen-Ramdial SA, Campbell AG (2014). Reimagining the pipeline: advancing STEM diversity, persistence, and success. Bioscience.

[27] Acosta D, Olsen P (2006). Meeting the needs of regional minority groups: the University of Washington’s programs to increase the American Indian and Alaskan native physician workforce. Acad Med.

[28] Vick AD, Baugh A, Lambert J (2018). Levers of change: a review of contemporary interventions to enhance diversity in medical schools in the USA. Adv Med Educ Pract.

[29] Glazer G, Tobias B, Mentzel T (2018). Increasing healthcare workforce diversity: urban universities as catalysts for change. J Prof Nurs.

[30] Ballejos MP, Olsen P, Price-Johnson T (2018). Recruiting American Indian/Alaska Native students to medical school: a multi-institutional alliance in the U.S. Southwest. Acad Med.

[31] Deas D, Pisano E, Mainous A (2012). Improving diversity through strategic planning: a 10-year (2002–2012) experience at the Medical University of South Carolina. Acad Med.

[32] Nair N, Marciscano AE, Vivar KL (2011). Introduction to the medical professions through an innovative medical student-run pipeline program. J Natl Med Assoc.

[33] Cooper RA (2003). Impact of trends in primary, secondary, and postsecondary education on applications to medical school. II: considerations of race, ethnicity, and income. Acad Med.

[34] Edlow BL, Hamilton K, Hamilton RH (2007). Teaching about the brain and reaching the community: undergraduates in the pipeline neuroscience program at the University of Pennsylvania. J Undergrad Neurosci Educ.

[35] Brodt E, Empey A, Mayinger P (2019). Shifting the tide: innovative strategies to develop an American Indian/Alaska Native physician workforce. Hawaii J Health Soc Welf.

[36] Derck J, Zahn K, Finks JF (2016). Doctors of tomorrow: an innovative curriculum connecting underrepresented minority high school students to medical school. Educ Health Abingdon.

[37] Fernandez-Repollet E, Locatis C (2018). Effects of summer internship and follow-up distance mentoring programs on middle and high school student perceptions and interest in health careers. BMC Med Educ.

[38] Crews DC, Wilson KL, Sohn J (2020). Helping scholars overcome socioeconomic barriers to medical and biomedical careers: creating a pipeline initiative. Teach Learn Med.

[39] Butts GC, Hurd Y, Palermo A-GS (2012). Role of institutional climate in fostering diversity in biomedical research workforce: a case study. Mt Sinai J Med N Y.

[40] Kana LA, Noronha C, Diamond S (2020). Experiential-learning opportunities enhance engagement in pipeline program: a qualitative study of the Doctors of Tomorrow Summer Internship Program. J Natl Med Assoc.

[41] Mains TE, Wilcox MV, Wright SM (2016). Medical education resources initiative for teens program in Baltimore: a model pipeline program built on four pillars. Educ Health Abingdon.

[42] Minhas PS, Kim N, Myers J (2018). Immersion medicine programme for secondary students. Clin Teach.

[43] Phillips KW, Liljenquist KA, Neale MA (2009). Is the pain worth the gain? The advantages and liabilities of agreeing with socially distinct newcomers. Pers Soc Psychol Bull.

[44] Fincher RM, Sykes-Brown W, Allen-Noble R (2002). Health science learning academy: a successful “pipeline” educational program for high school students. Acad Med.

[45] Patel SI, Rodríguez P, Gonzales RJ (2015). The implementation of an innovative high school mentoring program designed to enhance diversity and provide a pathway for future careers in healthcare related fields. J Racial Ethn Health Disparities.

[46] Muppala VR, Prakash N (2021). Promoting physician diversity through medical student led outreach and pipeline programs. J Natl Med Assoc.

[47] Toney M (2012). The long, winding road: one university’s quest for minority health care professionals and services. Acad Med.

[48] Burgos JL, Yee D, Csordas T (2015). Supporting the minority physician pipeline: providing global health experiences to undergraduate students in the United States-Mexico border region. Med Educ Online.

[49] Fritz CD, Press VG, Nabers D (2016). SEALS: an innovative pipeline program targeting obstacles to diversity in the physician workforce. J Racial Ethn Health Disparities.

[50] Stewart KA, Brown SL, Wrensford G (2020). Creating a comprehensive approach to exposing underrepresented pre-health professions students to clinical medicine and health research. J Natl Med Assoc.

[51] Campbell KM, Rodríguez JE (2019). Addressing the minority tax: perspectives from two diversity leaders on building minority faculty success in academic medicine. Acad Med.

[52] Thomson WA, Ferry PG, King JE (2003). Increasing access to medical education for students from medically underserved communities: one program’s success. Acad Med J Assoc Am Med Coll.

[53] Thomson WA, Ferry P, King J (2010). A baccalaureate-MD program for students from medically underserved communities: 15-year outcomes. Acad Med.

[54] Talamantes E, Mangione CM, Gonzalez K (2014). Community college pathways: improving the U.S. physician workforce pipeline. Acad Med.

[55] McFarland J, Pape-Lindstrom P (2016). The pipeline of physiology courses in community colleges: to university, medical school, and beyond. Adv Physiol Educ.

[56] Metz AM (2017). Medical school outcomes, primary care specialty choice, and practice in medically underserved areas by physician alumni of MEDPREP, a postbaccalaureate premedical program for underrepresented and disadvantaged students. Teach Learn Med.

[57] Judd NL, Sing PM (2001). Imi Ho’ola post-baccalaureate program: recruitment, retention, and graduation of Asian American and Pacific Islander students in medicine. Pac Health Dialog.

[58] Andriole DA, McDougle L, Bardo HR (2015). Postbaccalaureate premedical programs to promote physician-workforce diversity. J Best Pract Health Prof Divers Res Educ Policy.

[59] DeCarvalho H, Lindner I, Sengupta A (2018). Enhancing medical student diversity through a premedical program: a Caribbean school case study. Educ Health Abingdon.

[60] Phillips JL, Harris TB, Ihedigbo KM (2012). Saturday morning science programs: a model to increase diversity in the biosciences. J Natl Med Assoc.

[61] Butler BM (2011). Shifting patterns in the premedical education of African Americans and the role of the HBCU. J Afr Am Stud.

[62] Rodríguez JE, López IA, Campbell KM (2017). The role of historically black college and university medical schools in academic medicine. J Health Care Poor Underserved.

[63] Gasman M, Smith T, Ye C (2017). HBCUs and the production of doctors. AIMS Public Health.

[64] Young ME, Thomas A, Varpio L (2017). Facilitating admissions of diverse students: a six-point, evidence-informed framework for pipeline and program development. Perspect Med Educ.

[65] Grumbach K (2011). Commentary: Adopting postbaccalaureate premedical programs to enhance physician workforce diversity. Acad Med.

[66] Curtis E, Wikaire E, Stokes K (2012). Addressing indigenous health workforce inequities: a literature review exploring “best” practice for recruitment into tertiary health programmes. Int J Equity Health.

[67] Schellinger J, Cable K, Bond M (2020). The medical library as a component of a medical school outreach experience. Med Ref Serv Q.

[68] Prunuske A, Wilson J, Walls M (2016). Efforts at broadening participation in the sciences: an examination of the mentoring experiences of students from underrepresented groups. CBE Life Sci Educ.

[69] Yehia BR, Cronholm PF, Wilson N (2014). Mentorship and pursuit of academic medicine careers: a mixed methods study of residents from diverse backgrounds. BMC Med Educ.

[70] Pritchett EN, Pandya AG, Ferguson NN (2018). Diversity in dermatology: roadmap for improvement. J Am Acad Dermatol.

[71] Nivet MA, Berlin A (2014). Workforce diversity and community-responsive health-care institutions. Public Health Rep.

