# Supplementary material for: Physician Pipeline and Pathway Programs: An Evidence-based Guide to Best Practices for Diversity, Equity, and Inclusion from the Council of Residency Directors in Emergency Medicine
Source: West J Emerg Med. 2022 Jul 1;23(4):514–24. doi: 10.5811/westjem.2022.2.54875 (PMC9391003; doi:10.5811/westjem.2022.2.54875)
Supplement: Supplementary file 1 [file wjem-23-514-s001.docx]

**APPENDIX. Target Population, Participant Selection Criteria, and Application Components**

| **Explicitly Stated or Implied Target Racial/Ethnic Group** |  |
| --- | --- |
| American Indian/Alaskan Native (AIAN) | Acosta 2006; Bellejos 2018; Brodt 2019; Curtis 2012; Smith 2009; Prunuske 2016 |
| Black/African-American | Derck 2016; Edlow 2007; Fincher 2002; Smith 2009 |
| LatinX | Burgos 2015; Derck 2016; Edlow 2007; Fernandez 2018; Thomson 2003; Smith 2009 |
| Underrepresented Asian American and Pacific Islander | Judd 2001 |
| UIM or racial/ethnic minorities, not otherwise specified | Butts 2012; Crews 2020; Campbell 2019; Deas 2012; Toney 2012; Stewart 2020; Roche 2020; Phillips 2012; Prunuske 2016; Patel 2015; Muppala 2020; Minhas 2018; Metz 2017; Judd 2001; Fritz 2016; Andriole 2015 |
| **“Disadvantaged”, “underresourced”, and “underserved” backgrounds and communities** | Acosta 2006; Crews 2020; Deas 2012; Thomson 2003; Stewart 2020; Patel 2015; Judd 2001; Andriole 2015 |
| First-generation college student or “educationally disadvantaged” | Burgos 2015; Butts 2012; Crews 2020; Stewart 2020; Smith 2009; Prunuske 2016; Patel 2015; Mains 2016; Andriole 2015 |
| Socioeconomically disadvantaged; household income <200% of the federal poverty level | Burgos 2015; Butts 2012; Crews 2020; Stewart 2020; Schellinger 2020; Prunuske 2016; Minhas 2018; Metz 2017; Mains 2016; Andriole 2015 |
| Single-parent household | Crews 2020 |
| From rural or inner city community | Campbell 2019; Smith 2009 |
| Attending and “underresourced” high school or high school where majority of students live in for low income households | Crews 2020; Metz 2017 |
| Grew up in community with food deserts | Metz 2017 |
| Grew up in a health provider shortage area (HPSA) | Metz 2017 |
| **Interested in a health career or health disparities** | Acosta 2006; Bellejos 2018; Brodt 2019; Fincher 2002; Stewart 2020; Smith 2009; Roche 2020; Patel 2015; Muppala 2020; Fritz 2016 |
| Prior healthcare experience | Burgos 2015 |
| Selected from applicants who were not admitted to the medical school (Post-Baccalaureate Programs Only) | Campbell 2019; Deas 2012; DeCarvalho 2018; Andriole 2015 |
| Personal statement/essay | Crews 2020; Derck 2016; Stewart 2020; Schellinger 2020; Roche 2020; Patel 2015; Mains 2016; Kana 2020; Fritz 2016 |
| **Potential future academic success** | Minhas 2018; Andriole 2015 |
| Demonstrated prior academic success (GPA, standardized test scores, etc.) or in good academic standing | Crews 2020; Derck 2016; Thomson 2003; Stewart 2020; Smith 2009; Schellinger 2020; Roche 2020; Metz 2017; Kana 2020; Fritz 2016 |
| Motivation to succeed | Crews 2020 |
| Recommendation | Crews 2020; Thomson 2003; Stewart 2020; Schellinger 2020; Roche 2020; Metz 2017; Mains 2016; Kana 2020; Fritz 2016 |
| Identified by teachers or advisors as having an “untapped passion for learning.” | Crews 2020 |
| **Skills (i.e. computer, language, fundraising, writing, etc.)** | Burgos 2015 |
